# My Old House (Poetry)

**Published:** 1881-03

**Authors:** Z. P. Ormsby

**Affiliations:** Fayette, Me.


### MY OLD HOUSE.

Written by Mrs. Z. P. Ormsby, of Fayette, Me., on the 85th anniversary of her birthday.

I hail once more my natal day,
Still in my tenement of clay,
With many favors blest ;
And He who placed the structure here
Can prop it up another year,
If He should think it best.

Long has it stood through snows and rains,
And braved life's fearful hurricanes,
While many stronger fell.
The reason why we cannot see,
But what to us seems mystery
The Builder knows full well.

But now 'tis weather-worn and old ;
The summer's heat and winter's cold
Pierce through the walls and roof.
'Tis like a garment, so worn out,
To mend, there seems no whereabout,
So gone is warp and woof.

The tottering pillars all are weak ;
The poor old rusty hinges creak ;
The windows, too, are dim.
Those slight discomforts we'll let pass,
For, looking darkly through a glass,
We catch a hopeful gleam.

Nature and reason tell us all
This shattered frame ere long must fall,
When, where, or how, is all unknown ;
We'll leave that to the Architect,
And trust His wisdom to direct
The taking of it down.

And when you see it prostrate lie,
Let not a tear bedim the eye ;
The tenant is not here.
But just beyond time's little space
She finds some quiet resting-place,
No more to date her year.

And though she walks with you no more,
The world will move just as before ;
'Tis meet it should be so.
Let each his house in order set,
That he may leave without regret,
Whenever called to go.

